# Local transmission of chikungunya in Rome and the Lazio region, Italy

**DOI:** 10.1371/journal.pone.0208896

**Published:** 2018-12-21

**Authors:** Francesco Vairo, Alessia Mammone, Simone Lanini, Emanuele Nicastri, Concetta Castilletti, Fabrizio Carletti, Vincenzo Puro, Domenico Di Lallo, Vincenzo Panella, Donatella Varrenti, Paola Scaramozzino, Antonino di Caro, Paola Scognamiglio, Maria Rosaria Capobianchi, Giuseppe Ippolito, Chikungunya Lazio Outbreak Group

**Affiliations:** 1 Regional Service for Surveillance and Control of Infectious Diseases (SERESMI), National Institute for Infectious Diseases “Lazzaro Spallanzani” IRCCS, Rome, Italy; 2 National Institute for Infectious Diseases “Lazzaro Spallanzani” IRCCS, Rome, Italy; 3 Direzione Regionale Salute e Politiche Sociali, Regione Lazio, Rome, Italy; 4 Local Health Authority Roma 6, Albano Laziale, Italy; 5 Istituto Zooprofilattico Sperimentale del Lazio e della Toscana “M. Aleandri”, Rome, Italy; CEA, FRANCE

## Abstract

On September 7, 2017, three potentially autochthonous cases of chikungunya were notified in the Lazio region. An Outbreak investigation based on established surveillance system data and molecular analysis of viral variant(s) were conducted. Epidemiological analysis suggested the occurrence of 3 main foci of local transmission. The major focus involved 317 cases with epidemiological link with the area of Anzio. The other two foci occurred in Rome (80 cases) and Latina (8 cases). Cumulative incidence in Anzio and Latina were 331.4 and 7.13 per 100,000 residents, respectively. Cumulative incidences ranged from 1.4 to 14.3/100,000 residents in Rome. This is the first report of a chikungunya outbreak involving a densely populated urban area in a western country. The outbreak probably started in Anzio, spread by continuity to neighbouring villages, and then to the metropolitan area of Rome and to the Latina area favoured by the touristic nature of the Anzio area.

## Introduction

Chikungunya virus (CHIKV) is an alphavirus associated with a mosquito-borne infection usually causing a systemic self-limiting disease with a wide range of clinical presentations. Chikungunya disease generally includes fever, moderate to severe arthralgia and often maculopapular exanthema. About 15% of CHIKV infections may be asymptomatic [1].

CHIKV was firstly described during an outbreak in southern Tanzania in 1952–53 [2]. Since then the virus was found as one of the main cause of mosquito-borne infection in tropical and subtropical regions where it caused epidemics involving millions of people [3,4]. Following the expansion of the geographical distribution of its mosquito vectors (mainly *Aedes aegypti* and Aedes *albopictus*), [5] CHIKV has spread beyond its original tropical locations (Africa and the Indian subcontinent) and, since recently, it has become an emerging issue in temperate regions of Northern Hemisphere. In particular small autochthonous outbreaks occurred, as the consequence of spill over from large ongoing transmission in tropical areas, in continental Europe including Italy in 2007, and in France in 2010, in 2014, and eventually in 201 [6–9].

On September 7, 2017, the Lazio Regional Service for Surveillance and Control of Infectious Diseases (SERESMI) reported to the Italian Ministry of Health (MoH) a cluster of three autochthonous cases of CHIKV infection in Anzio, a coastal town devoted to internal tourism, 1 hour driving from Rome.

Here we report the comprehensive results of the investigations of the largest CHIKV outbreak in a temperate climate with the involvement of a highly populated urban area.

## Methods

### Study design

Outbreak investigation based on an established surveillance system data and molecular analysis of viral variant(s).

### Setting

Lazio is the second most populated Region of Italy (5,898,124 residents). The 48.72% (N = 2,873,486) of Lazio inhabitants live in the Urban area of Rome (which is divided in 15 administrative districts “*municipi”*; median inhabitants 180,896 IQR 155,201–231,037). All other people live in the remaining 346 municipalities (*comuni*), mainly towns (median inhabitants 2,619 IQR 1,096–8,094). Anzio (54,311 inhabitants) is a town located 62 Km southern of Rome on the coastline. Due to the proximity to Rome, many residential areas have been developed over the last 40 years to host commuters from Rome that spend the summer in their own beach house.

Since 2015, Lazio Regional Health Authority has implemented an integrated surveillance system for chikungunya, dengue and zika, based on the national plan for surveillance and control of arboviruses transmitted by *Aedes* mosquito.

### Participants and case definition

Following the notification of the autochthonous cluster, the surveillance system was strengthen through the dissemination of a dedicated case definition in order to increase the sensitivity, promptly trace contacts and early identify the affected areas throughout the Region.

All people living in Lazio and matching one of the following definitions:

- ***Suspected case***: a person that either A) had a sudden onset of fever and joint pain without history of travel to an endemic country in the 15 days before symptoms’ onset; or B) reporting an epidemiological link with a probable/confirmed case. Epidemiological link was defined as being a relative, living in the same household or being a neighbor (living in radius of 200m from the case household) of a confirmed/probable case;
- ***Person under investigation***: any suspected case A) without an available serological and/or molecular test performed or B) with a unique determination of detectable anti-CHIKV IgG;
- ***Probable case***: any suspected case that tested positive for anti- CHIKV IgM on a single serum sample;
- ***Confirmed case***: any suspected case that: A) tested positive for CHIKV PCR or B) tested positive for anti- CHIKV IgM on a single serum sample confirmed by sero-neutralization or C) seroconverted from negative to positive or D) showed a fourfold increase of Ig titer in two subsequent samples taken at least 2 weeks apart;
- ***Non case***: a person that either: A) had undetectable IgM, IgG and PCR if tested ≤10 days since symptoms onset; B) had undetectable IgM and IgG if tested >10 days since symptoms onset.

An imported case was defined as a probable or confirmed case with a history of travel to an endemic country during the 15 days before the onset of symptoms.

### Data and sample collection

Any subject responding to the definition of suspected case was notified to SERESMI within 24 hours and samples sent to the Regional Reference Laboratory. In Anzio, a door-to-door active case finding was implemented in the houses and apartments located inside a 200m radius from a probable or confirmed case. Any subject reporting fever and joint pain during the previous 5 months was notified as suspected case and tested. We collected data on patients’ age, sex, place of living, time of symptoms’ onset, history of travel within Italy or abroad during the 15 days before symptoms’ onset. Daily situational reports (epidemic curves, geolocalization) were provided to the MoH and the National Blood Center in order to promptly implement the related control measures.

### Virology diagnostics

CHIKV diagnosis was based on the detection of the viral genome by real-time RT-PCR and virus-specific antibodies by serologic tests on serum or plasma samples. For the qualitative detection of CHIKV specific RNA a commercial real-time RT-PCR kit (RealStar Chikungunya RT-PCR Kit 2.0, Altona Diagnostics GmbH, Hamburg, Germany) was used. Serologic tests for the detection of CHIKV specific IgG and IgM were performed using indirect immune fluorescence assay (IFA) (Anti-Chikungunya Virus IIFT Euroimmun AG, Germany). Neutralization assay was performed according to Lindsey HS et al [10].

### Molecular investigation

The molecular characterization of E1 was performed for confirmatory purpose on samples collected at various time points (from the first three notified cases, from three cases in mid-September, from two cases at mid-October) [11]. The complete genome sequencing of the isolate obtained from the acute serum sample of a patient living in Anzio, collected on September 11, 2017, was performed. A 11.604 nt long sequence was amplified in 23 overlapping RT-PCR amplicons and Sanger sequenced as described elsewhere (isolate CHIKV/ITA/Lazio-INMI1-2017, GenBank accession number: MG049915) [12]. The phylogenetic tree was built using Maximum-Likelihood method based on the full-length genome sequences of 46 isolates, including the CHIKV/ITA/Lazio-INMI1-2017 sequence, representing the 3 major described CHIKV lineages: ECSA (including the Indian Ocean lineage, n = 29), Asia-Caribbean (n = 12), and West Africa (n = 4). Evolutionary distances were computed using the General Time Reversible model (GTR). The alphavirus O’nyong’nyong was used as an outgroup. Bootstraps were generated using 1,000 replicates. In addition, the CHIKV/ITA/Lazio-INMI1-2017 sequence was compared to the reference sequence S27, the African prototype strain isolated in 1953 [13].

### Statistical analysis

Statistical analyses were performed using STATA 13.1. Cumulative incidence, considering the residing population as at risk population, was used as measure of frequency of morbidity. Papulation census from the year 2016 was used (http://demo.istat.it/). Spatial distribution of cases (probable and confirmed) was graphically reported as cumulative incidence per 100,000 residents and plotted on a choropleth map using the 361 administrative area of Lazio (i.e. 346 towns of Lazio and the 15 *municipia* of Rome).

### Ethics statement

According to REGULATION (EU) 2016/679 OF THE EUROPEAN PARLIAMENT AND OF THE COUNCIL of 27 April 2016 on the protection of natural persons with regard to the processing of personal data and on the free movement of such data, and repealing Directive 95/46/EC (General Data Protection Regulation). Informed consent was not obtained from the participants because of the public health emergency during an infectious disease outbreak. All data contained in the manuscript were obtained during the epidemiological investigation as an institutional duty of the Latium Regional Health Authority, in order to identify/contain the ongoing outbreak, to provide recommendations on control measures and to avert complications in infected subjects. The approval of the National Institute for Infectious Diseases Spallanzani’s Institutional Review Board was not required for the same reasons. Patients never underwent individual intervention for the purposes of this study but only according to their needs and clinical judgment. Data have been analyzed anonymously.

## Results

### Epidemiological investigation

**Fig 1:**
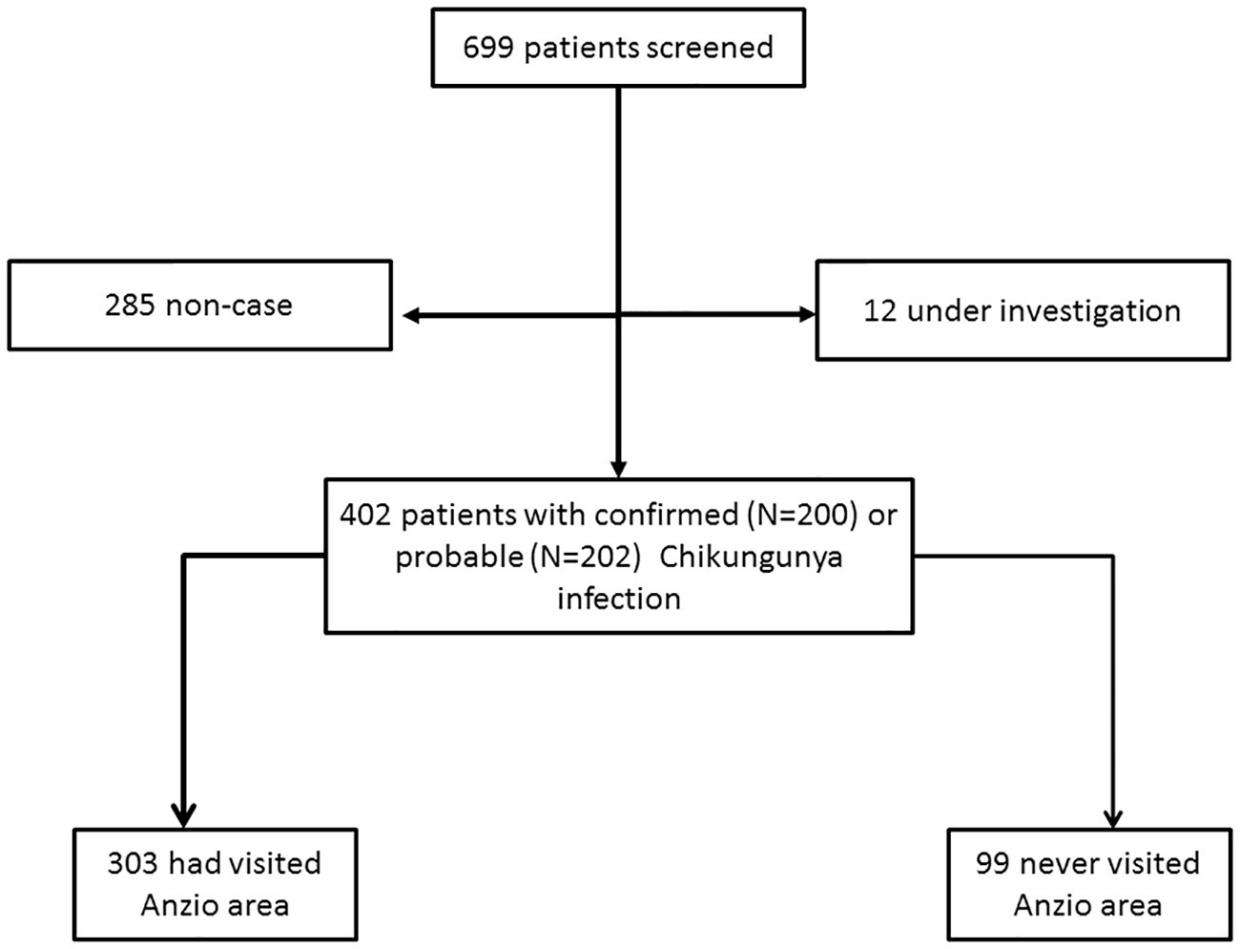
Case definition flow-chart.

Between January 1, 2017 and January 31, 2018, 823 suspected cases were reported to the regional surveillance system. Of them, 699 (84.9%) responded to the case definition for autochthonous chikungunya suspected cases. Of these, 285 (40.8%) were non cases and 12 (1.7%) remained under investigation. Of the remaining 402 (57.5%) cases, 200 (49.8%) were classified as confirmed cases and 202 (50.2%) as probable cases. Fig 1 reports case definition flowchart.

**Fig 2:**
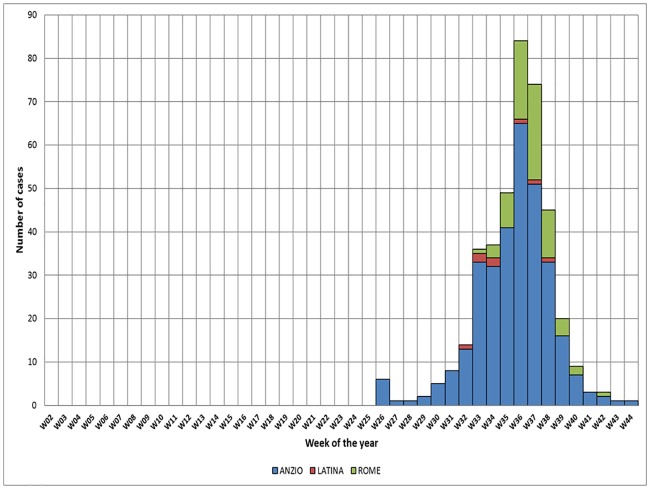
Epidemic curve of autochthonous CHIKV cases by probable place of transmission. **NOTE**. Total number of cases reported is 399. Two cases with no epidemiological link with Anzio were residing in two towns in Rome province and one case had not date of symptoms’ onset available. Anzio, cases residing in Anzio or with probable transmission in Anzio; Rome, cases with no epidemiological link with Anzio and with probable transmission in Rome; Latina, cases with no epidemiological link with Anzio and with probable transmission in Latina. Probable place of transmission is defined as the city where the case was continuously residing for the 15 days before the onset of symptoms.

Fig 2 shows the epidemiological curve of cases by possible place of transmission. The first three notified cases had symptoms’ onset on August 5, 11 and 25 and occurred among people living in a residential area, made of single-family detached houses, in Anzio. Following the strengthen of the surveillance system and the door-to-door case finding, the epidemiological investigation suggested the occurrence of three mainfoci of local transmission. The largest focus, involving 317 persons with a direct link with the town of Anzio and its surrounding area, occurred between week 26 and week 42. The earliest symptoms’ onsets were June 26 (2 cases) and 27(2 cases). The other two foci occurred in people who lived in Rome and Latina and had no link with the area of Anzio. In Latina, the second largest urban area of Lazio, 8 cases occurred between week 32 and week 38. The first case was notified on September 12 and earliest reported symptoms’ onset was August 13. The focus of Rome involved 80 cases in the metropolitan area and 2 cases in two towns in the Province between week 33 and week 42. The first case was notified on September 7 and the earliest reported date of symptoms’ onset was August 20. The last symptoms’ onset was November 5 in a case reported from Anzio.

Between January 1, 2017 and January 31, 2018, four imported cases of CHIKV were notified. Only two of them had symptoms’ onset before the notification of the outbreak. One imported case (probable) was notified in January in a traveler returning from Venezuela with symptoms’ onset on January 10 and returning date on January 22. The other case (confirmed) was notified on July 29 with symptoms’ onset on July 27, one day after the return from Cameroon. The molecular characterization of E1 showed a sub-lineage of the ECSA clustering with lineage from Central African Region.

**Fig 3:**
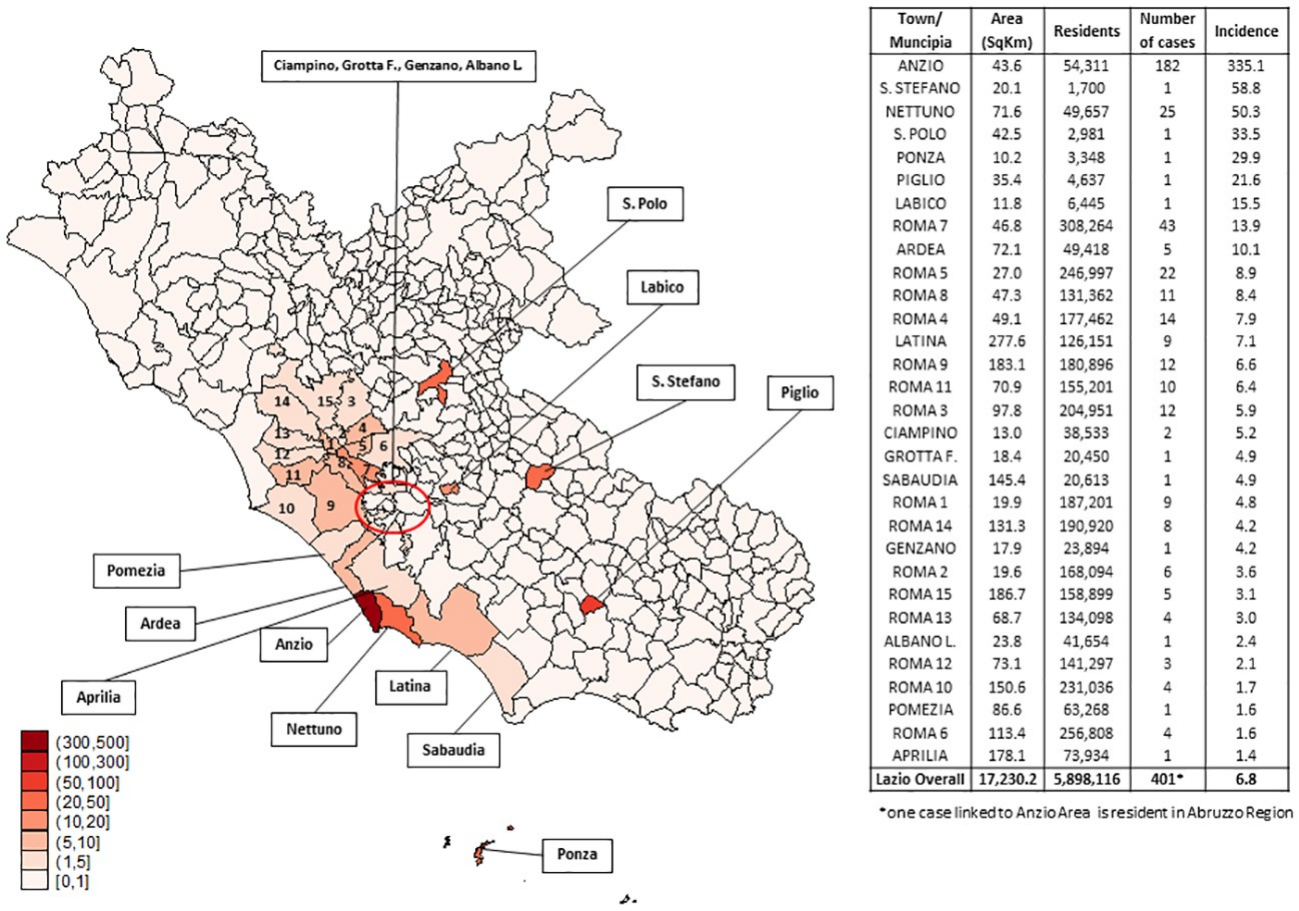
Geographical distribution of cases according to incidence (per 100.000 inhabitants). **NOTE**. Incidence has been calculated considering the place of living of the reported cases and not the epidemiological link (history of travel during the 15 days before the symptoms’ onset). *one case linked to Anzio Area is resident in Abruzzo Region.

Following the cases in Anzio, incident cases occurred in 16 cities of Lazio and, by the week 33 throughout the 15 districts of Rome. Fig 3 shows the spread of the infection by contiguity across neighboring villages and Rome. Distribution of cumulative incidence (Fig 3) shows higher values in two coastal towns where first cases had occurred (excluding towns were only 1 case occurred): Anzio (N = 182 cases; 335.1 per 100,000 residents) and Nettuno (N = 25 cases; 50.4 per 100,000 residents). The cumulative incidence in Rome shows a variable distribution by *municipia* ranging from 1.6 to 13.9 per 100,000 residents, in the different districts. Latina reported a cumulative incidence of 7.1 per 100,000 residents. The cumulative incidence in the whole region was 6.8 cases/100,000.

### Clinical presentation

The distribution of demographic and clinical characteristics of the probable or confirmed cases is shown in Table 1. Among the 402 probable/confirmed cases, the infection was equally distributed between gender with a slightly higher proportion among males. Median age was 55 years (IQR 40.0–67.5) and the 56.2% of cases were older than 50 years. The 96·8% (389/402) of cases reported fever, 95.8% (385/402) reported joint pain, 62.9% (253/402) developed a skin rash and 39.6% (159/402) developed arthritis. Sixty-nine suspected cases did not report fever and were tested because close contacts (family/household/neighbor) of a confirmed case. Among these, 13 were confirmed/probable cases and had an epidemiological link with a case in Anzio [8] or in Rome [5] and 56 were classified as non-cases. Thirty-five (9.4%) cases were admitted to hospital. One death was reported in a 77 years old man with underlying cardiovascular disease who was diagnosed with chikungunya infection after an admission for a stroke.

**Table 1:** Demographic and clinical characteristics of the 402 probable or confirmed cases.

Demographic and clinical characteristics		Number of cases (%)
**Age, years**	**0–34**	81 (20.2%)
	**35–50**	95 (23.6%)
	**51–64**	99 (24.6%)
	**>65**	127 (31.6%)
**Sex**	**Male**	184 (45.8%)
	**Female**	218 (54.2%)
**Body temperature**	**<37.8 °C**	13 (3.2%)
	**> = 37.8 °C**	389 (96.8%)
**Hospital admission**	**No**	367 (90.6%)
	**Yes**	35 (9.4%)
**Arthritis**	**No**	243 (60.4%)
	**Yes**	159 (39.6%)
**Headache**	**No**	196 (48.8%)
	**Yes**	206 (51.2%)
**Myalgia**	**No**	148 (36.8%)
	**Yes**	254 (63.2%)
**Retro orbital pain**	**No**	353 (87.8%)
	**Yes**	49 (12.2%)
**Conjuntivitis**	**No**	342 (85.1%)
	**Yes**	60 (14.9%)
**Rash**	**No**	149 (37.1%)
	**Yes**	253 (62.9%)
**Asthenia**	**No**	91 (22.6%)
	**Yes**	311 (77.4%)
**Arthralgia**	**No**	17 (4.2%)
	**Yes**	385 (95.8%)

### Molecular characterization

**Fig 4:**
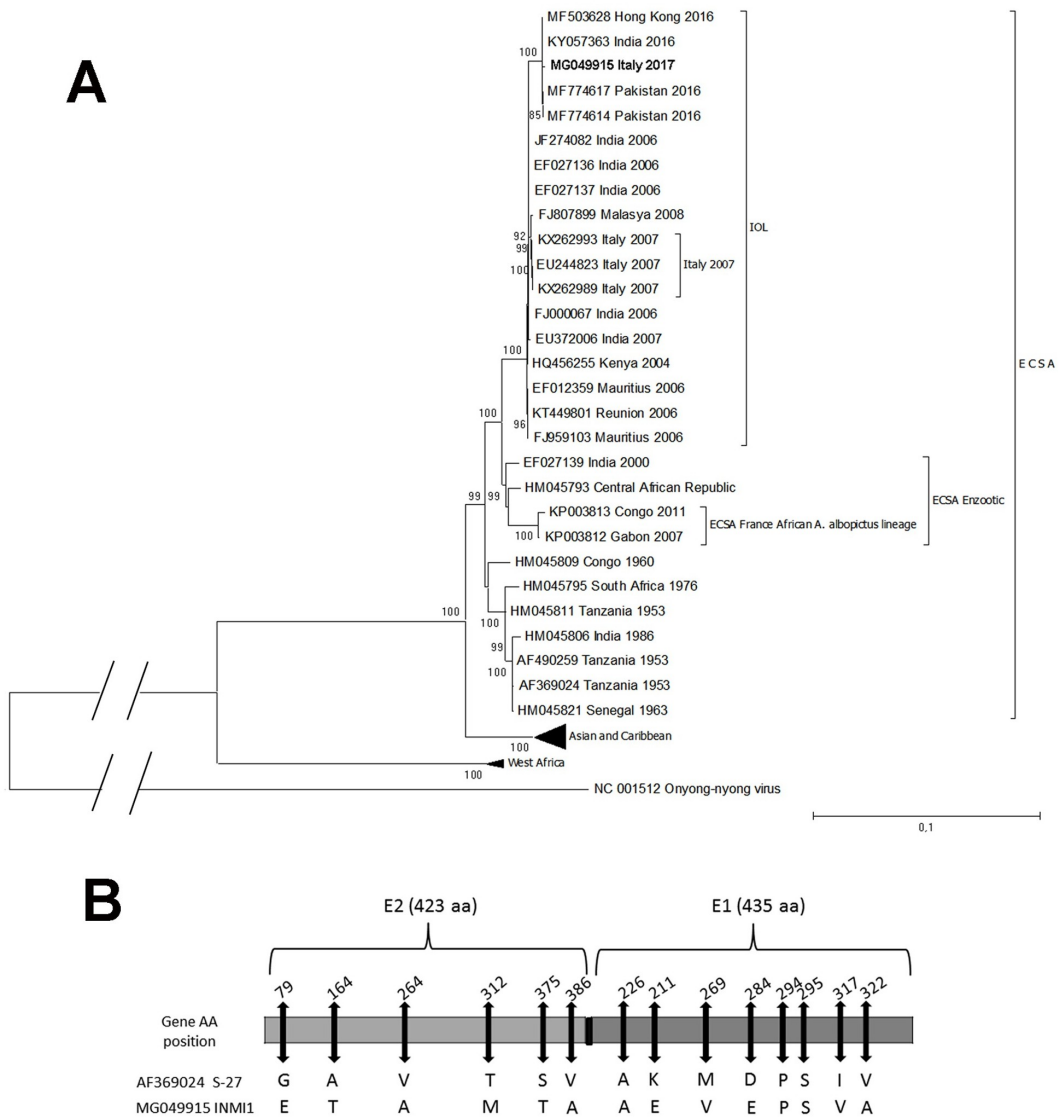
Phylogenetic tree of the complete genome sequence (A) and polymorphisms in E1 and E2 envelope glycoproteins (B). A) Phylogenetic tree of the complete genome sequence of the isolate CHIKV/ITA/Lazio-INMI1-2017 obtained from the current outbreak, Lazio region, Italy. The Maximum-likelihood phylogenetic tree was built with the complete genome sequence of the isolate CHIKV/ITA/Lazio-INMI1-2017 (outlined in bold characters) in the context of whole genome sequences representing the 3 major described CHIKV lineages: ECSA (including the Indian Ocean lineage), Asia and Caribbean, and West Africa. These sequences are indicated with their accession number, geographic origin and year of sampling. Asian and Caribbean and West Africa sequences are collapsed to increase the clarity of the figure. Bootstraps were generated using 1,000 replicates; only those >80 are shown. The bar represents genetic distance (substitutions per nucleotide position). B) Polymorphisms in E1 and E2 envelope glycoproteins of CHIKV/ITA/Lazio-INMI1-2017, at positions considered relevant for vector adaptation.

Preliminary molecular characterization, based on partial sequence of the E1, indicated that the sequences from samples collected at different time points of the outbreak were all identical, and belonged to the Indian Ocean subclade (IOL) of the ECSA lineage, as observed for the sequences from the 2007 outbreak in Italy. In Fig 4, phylogenetic tree of the complete genome sequence of the isolate CHIKV/ITA/Lazio-INMI1-2017 is shown in the context of sequences representing the 3 major described CHIKV lineages: ECSA, Asia and Caribbean, and West Africa. As can be seen, CHIKV/ITA/Lazio-INMI1-2017 is located on a branch of the IOL sublineage that is distinct from that comprising of the 2007 Italian autochthonous sequences, and clusters with recent strains originating from Pakistan and with an isolate obtained in China, from a traveler returning from India. Considering E1 and E2 polymorphisms at sites considered relevant for vector fitness, CHIKV/ITA/Lazio-INMI1-2017 lacks the adaptive mutation A226V in E1, and, more generally, shows the genetic signature of strains using *Ae*. *aegypti* as preferential vector (Fig 4B) [12].

## Discussion

This is the first report of a CHIKV outbreak involving a highly populated urban area in a western country. Based on the epidemic curve and on the incidence map, the outbreak may have started in the area of Anzio and spread by continuity to the neighboring villages and then to the Urban area of Rome and to the city of Latina favored by the touristic nature of the Anzio area. Anzio is one of the most popular location for internal tourism of people living in Rome who spend summer time in their second houses on the coast commuting to Rome on daily basis. The touristic nature of Anzio could explain also secondary outbreak of CHIKV in September and October 2017, in Guardavalle Marina, a small village of 2,346 inhabitants in the province of Catanzaro (CZ), Calabria region (Southern Italy) [14].

The cumulative incidence was higher in coastal and rural areas compared to that reported in Rome as also predicted by transmission models based on mosquitoes abundance and biting rate [15]. The cumulative incidence in Anzio and Rome area is lower than the incidence in the 2007 outbreak in Castiglione di Cervia (5.4%) and in Castiglione di Ravenna (2.5%) and the incidence in tropical areas [16–18] but higher than the incidence observed during the 2017 event in France in Le Cannet-des-Maures (9 cases out of 4.500 inhabitants) [9].

The median age of the patients was 55 years (IQR 40.0–67.5). Similar age profile was reported in the 2007 outbreak in Italy (median 60 years) [6] and in 2017 outbreak in France (range 33–77 years) [9]. The increasing prevalence rate of CHIKV infection with increasing age is consistent with other outbreaks, which report lower seroprevalence in children in Italy, in Bagan Panchor, Malaysia, in Mayotte, Indian Ocean and in Managua, Nicaragua [19–22]. Age is a proxy-factor for specific behaviors that cause higher exposure to *Ae*. *albopictus* bites (i.e., staying outdoors during daytime) or less tendency toward individual protection (i.e., use of insect repellents) in elder people. Elder people are also more likely to show symptoms and to access to the health structures than young people are [22].

Fever and joint pain, usually localized in both the arms and legs, is reported in 90% of patients [23]. Skin rash is usually reported in a variable proportion of cases (between 20% and 80%). The distribution of symptoms is similar to the one reported during the previous outbreak in Italy in 2007 [6] and during the 2017 outbreak in France [9]. The relative frequencies of fever, joint pain and rash were higher than those reported from the Pakistan 2016 outbreak (caused by the same clade), where fever and joint pain occurred in 85·9% and 88·4%, respectively, and rash occurred in only 29.1% of cases [24]. The case fatality rate was 2.5 per 1,000 clinical cases, lower than the one reported in the 2007 outbreak in Italy (0.5%) but consistent with those reported from la Reunion (1 death per 1,000 clinical cases) [6,25].

As reported by Carletti et al [12], the phylogenetic analysis shows that the virus involved in the current Lazio outbreak belongs to the East, Central, and South Africa (ECSA) clade, and clusters within the IOL. The sequences from the current outbreak are placed in a separate branch of the phylogenetic tree compared with the isolates from the 2007 Italian outbreak. Isolates from recent outbreaks in Pakistan and India are placed in the same branch, suggesting a more recent origin of the new epidemic strain. Given the molecular characterization of the strain and its phylogenetic characteristics, a possible introduction of the virus from the ongoing epidemic in Pakistan by travelers could be hypothesized [26]. This hypothesis is also supported by the seasonal synchronicity between Italy and India or Pakistan, as it happened also during the outbreak in Italy in 2007 [27]. The concurrent 2017 French outbreak showed a sub-lineage of the ECSA clustering with lineage from Central African Region and carrying the A226V mutation [9]. This excludes a possible link between the two outbreaks. Finally, the epidemiological and molecular characteristics of the imported cases prior to the outbreak rule out these cases as possible source of introduction. Of note, the characterization of E1 performed at various time points during the outbreak indicated a virtually complete identity of the virus sequences along the outbreak suggesting no additional introductions during the outbreak.

E1 sequences lack the A226V substitution associated with increased viral fitness in *Ae*. *Albopictus* [11]. The virus was isolated also from mosquitoes in Anzio area and showed the same clade and mutational patterns [28] of the human strain.

*Ae*. *albopictus* is disseminated through at least nine regions in Italy: Veneto, Lombardy, Emilia-Romagna, Liguria, Tuscany, Lazio, Piedmont, Campania, Sardinia [29] and Calabria [30].

The absence of the A226V may have had implication for transmission efficiency in the present outbreak, given that *Ae*. *Albopictus* is the unique competent vector circulating in Italy. This could explain the lower cumulative incidence compared with other urban outbreaks although the role of different background vector density or climate-dependent vector behavior cannot be excluded. The 2017 was characterized by an exceptional dry summer season. Despite the low cumulative incidence, this outbreak showed a very long duration (from June to November). Fig 4B shows a greater number of mutations that, considering E1 and E2 polymorphisms at sites relevant for vector fitness, provide the genetic signature of strains that use *Ae*. *aegypti* as preferential vector. The role of these mutations in the virus’ ability to better replicate and disseminate in *Ae*. *Albopictus* is still unclear. A comparison of the vector competence for the 2007 and 2017 Italian strains showed similar vector competence for both strains suggesting A226V could not be the sole responsible for ability of CHIKV to replicate in *Ae*. *Albopictus* [31]. Moreover, as already demonstrated for dengue and yellow fever [32], the genetic background of the autochtonous *Ae*. *Albopictus* could facilitate the competence for the virus despite the presence of the A226V mutation and should be further evaluated.

Vector control activities have been carried out by using insecticides for adult mosquitoes knock-down (space spraying with pyrethrines) and residual insecticides for resting mosquitoes (etofenprox based products) applied on vegetation. Preventive blood safety measures were also introduced in the different areas following the spread of the infection. Given the large and populated area of Rome and the consequences of a interruption of blood donations on the regional blood supply, a risk-benefit evaluation based on daily epidemiological data was performed [33].

The reappearance of CHIKV in Italy and the involvement of urban settings could have been influenced by several factors. The globalization of travel together with the global spread of CHIKV could facilitate the importation of the infection, as was the case in the 2007 outbreak [6]. The adaptation of the CHIKV to specific vector species though genome mutation is a key factor in the CKIKV spread. The influence of climate change on the vector’s climatic suitability put the Mediterranean region at high risk for CHIKV dissemination [34, 35].

The date of symptoms’ onset of first reported case was August 5, 2017 and the earliest symptoms’ onset was June 26. The surveillance system was able to detect the first autochthonous case only after the peak of the outbreak as it was in 2007 [6]. Early identification of autochthonous cases of chikungunya infection is the main challenge for the passive surveillance system in place. In Italy, the activity of the vector mosquitoes is mainly restricted to the summer season (usually between June and October) when outbreaks could be triggered by the arrival of imported cases from endemic areas and may result in secondary cases. This highlights the importance of an integrated surveillance system that should promptly identify autochthonous transmission. The integration of the passive surveillance with different surveillance tools (such as laboratory-based surveillance, syndromic surveillance, novel data stream) [36] combined with entomological surveillance should facilitate the detection, response and control of arboviruses spreading, including CHIKV.

Finally, information and training for medical professionals might be useful to favor early diagnosis and reporting of exotic viral diseases such as chikungunya.

## Supporting information

S1 File Database. This a minimum anomyzed data set is available. 0, Absent; 1, Present; F, Female; M, Male.
(XLSX)
